# An Intelligent Classification Method of Multisource Enterprise Financial Data Based on SAS Model

**DOI:** 10.1155/2022/8255091

**Published:** 2022-03-24

**Authors:** Xiuyan Xu

**Affiliations:** Xijing University, Xi'an, Shaanxi 710123, China

## Abstract

An enterprise is often faced with a large amount of financial information and data information. It is inefficient to rely solely on manual work, and the accuracy is difficult to guarantee. For the multisource data of corporate finance, it is more difficult for financial personnel to accurately analyze the connections between the data. For the multisource financial data of enterprise, this is also a time-consuming and laborious task for financial personnel. At the same time, it is difficult to find the correlation between multiple sources of data and then formulate financial data that guides the development of the enterprise. With the advancement of intelligent algorithms, an intelligent classification algorithm similar to the SAS model has emerged, which can realize the intelligent classification of enterprise financial multisource data and accurately predict the future development trend, which is extremely beneficial to the development and performance of the enterprise. This article mainly combines the financial intelligence classification model SAS with clustering and decision tree methods to classify the financial multisource information and uses the neural network method to carry out the future development trend of corporate finance. The research results show that the maximum error of enterprise financial classification after using the intelligent classification method is only 3.71% and that the forecast error of the future development trend of enterprise finance is only 1.77%. This is an acceptable error range, and this intelligent classification method is also greatly improving the efficiency of corporate financial management.

## 1. Introduction

The development of an enterprise is not only affected by product performance. As the economic system changes, more companies pay more and more attention to the development and fluctuations of corporate finance, which will reflect to a certain extent the operation of the company in each time period [1]. The status provides a reference for the next development plan. The product level and quality of an enterprise only determine the situation of the product itself, and the finance of the enterprise is an important reference for determining development decisions and price setting. Therefore, the development of an enterprise often requires a large number of financial personnel as the support for the development of the enterprise. For a small company, the efficiency of financial personnel can support the analysis and planning of the company and products, but for medium and large enterprises, this task is often difficult, and the efficiency of corporate financial personnel and human factors are often limited [2, 3]. At the same time, with the development of economic globalization, the financial data of enterprises is not just a simple single-input and single-output relationship. SAS model is an important classification and prediction model in the field of intelligent finance. Using SAS model can solve the classification and prediction problems of multisource financial data that are difficult to handle for enterprise financial personnel, which is convenient [4].

In the face of economic globalization and the prevailing economic era of e-commerce, the development of enterprises is bound to generate data and information from multiple channels [5]. Corporate financial information is a necessary means to guide the company long-term development and make changes in accordance with economic conditions [6]. For the development of an enterprise, it is often not enough to merely rely on the improvement of product quality and technological level. A large enterprise often has a large financial team. However, with the development of smart financial economy in recent years, smart algorithms have shown good performance and applicability in the financial field [7]. The intelligent financial classification method will not only improve the classification and processing of financial data, but it will also analyze the future financial trend of the enterprise based on the relationship between multisource data for reference by enterprise decision makers. This method also reduces the complicated business volume of the financial staff and improves the efficiency of the financial staff [8]. For multisource data in finance, it is difficult for financial personnel to discover the nonlinear correlations only by relying on their own knowledge level. Intelligent algorithms will assist humans to recognize these auxiliary nonlinear correlations [9], whether they are the trend of data changes or the relationship with time.

With the diversification of corporate financial data and the continuous advancement of intelligent classification methods, a lot of research on intelligent financial classification methods has been carried out in the field of corporate finance, and many excellent results have been also achieved [10]. Zhang et al. [11] used the firefly algorithm to optimize the support vector machine (SVM) algorithm and developed a new type of firefly support vector machine (FA-SVM) algorithm. The credit risk of supply chain finance was evaluated and verified, and the results showed that the algorithm they proposed has high accuracy in the financial field and can accurately find groups with lower credit. Chen et al. [12] studied the relationship between the Internet and corporate financing efficiency using the data envelopment analysis method and the slack based measure method (DEA-SBM). At the same time, they analyzed the role of corporate financing spillovers by means of spatial measurement and concluded that smart methods can help improve the financing rate of enterprises and accurately capture the spatial spillover rate. In response to the large workload and error-prone problems in current corporate financial revenue forecasting, Huang and Huang [13] proposed a support vector machine-based corporate financial revenue forecasting model, which is based on the method of the average mean square error, absolute error value (MAPE), and other quantitative parameters. The analysis and the conclusion show that the support vector machine method is suitable for enterprise financial revenue forecasting. Wang et al. [14] believe that smart methods can identify target risk rates and reduce risks in corporate crowdfunding tasks. They applied decision tree, logistic regression, support vector machine, and deep learning methods to compare risk identification, and the accuracy rate reached 92.3%. This is a beneficial way for Internet finance. Sang et al. [15] used genetic algorithm and BP neural network to predict and analyze the dynamic changes of financing services and commodity flows of small- and medium-sized enterprises. The accuracy rate of the BP neural network algorithm for the evaluation of the supply chain of small and medium enterprises has reached 89.3%. This is a relatively low accuracy for small and medium enterprises. The loss of bank interest rates makes it possible. Zhu et al. [16] combined two traditional machine learning methods, random subspace (RS) and MultiBoosting, to propose a hybrid enhanced machine learning model. The results show that this method has good feasibility and accuracy when dealing with small and medium samples in enterprises. Tubastuvi [17] believes that small- and medium-sized enterprises have many problems and that the problem of funding is particularly prominent. He studied the impact of loss-sharing PLS contracts on financing channels and used structural equation model SEM to classify the data. The conclusion shows that a higher PLS contract will promote the development and financing of small- and medium-sized enterprises. Li [18] studied the debt relationship in shipping companies, proposed an intelligent strategy model, and divided shipping companies' data into explicit and invisible forms. The results showed that this method is more efficient than the traditional shipping economic evaluation model. Zdravkovic et al. [19] integrated industrial Internet technology, distributed systems, cloud computing, and deep learning technology to study the application of artificial intelligence technology in enterprises. This method can realize the independent decision-making function of corporate finance.

From the above review of the development status of corporate finance, it can be seen that the current research on corporate finance mainly uses machine learning and other types of algorithms to study the trend of a single data source. This study analyzes the performance of intelligent classification of multisource data of corporate finance, and good intelligent classification makes sense for the business [20]. Intelligent financial classification will reduce the workload of financial staff and reduce the error rate of thinking. At the same time, financial staff will make full use of professional knowledge to make more advanced classification planning [21]. If an enterprise can well combine the advantages of smart finance and manual finance, the company will respond well to the development trend of the global economy, which is beneficial to the long-term development of an enterprise [22]. Moreover, the development of computer hardware and the advancement of intelligent classification algorithms at this stage provide more support for intelligent financial classification, such as the SAS model library [23, 24]. Decision trees, support vector machines, neural networks, etc. are all excellent classification models in the SAS model library, which are easy to build, and perform predictive analysis for intelligent financial classification and forecasting. SAS provides more convenience for the classification of smart finance.

This article is mainly divided into 5 sections to introduce the feasibility of intelligent financial classification. The first section introduces the current situation and necessity of the development of enterprise financial intelligence. The second section mainly introduces the significance of the development of intelligent financial classification for corporate performance and the source of the data set. The third section introduces the main algorithms and processes to realize the intelligent financial classification of enterprise performance. The fourth section explains the feasibility and accuracy of classification methods and forecasting methods in enterprise financial forecasting and classification. The fifth section is a summary of the feasibility analysis of enterprise financial intelligence classification tasks.

## 2. The Necessity and Data Source of SAS Model for Intelligent Classification of Enterprise Multisource Data

### 2.1. The Significance of Smart Financial Data Classification

With the development of economic globalization and the continuous development of the e-commerce economy, the economic system and business model of enterprises have undergone major changes compared to previous years [25]. Enterprise data also come from multiple sources. If only financial personnel are used to process cumbersome financial data, it will be difficult, and it is easy to produce certain wrong information. The SAS model library can provide a variety of intelligent forecasting algorithms for corporate financial analysis, and the construction is simple and efficient, such as decision trees, random forests, and neural network algorithms [26]. The sources of multisource financial data are different, and the characteristics of the data information are also different. Not only is corporate financial staff difficult and cumbersome to deal with, but it is also difficult to find the correlation between its multisource data and to use the data itself to predict the development trend of the corporate economy [27, 28]. Economic development is changing rapidly, which requires business managers to have insight into financial data and information and then make corresponding business development decisions [29]. The SAS model can find the correlation between these multisource data and provide new discoveries and clues for the financial staff [30]. Once the mapping relationship between multisource data is established through SAS, corporate financial personnel can use their own knowledge to further lock the relationship between multisource data and corporate products, which is a very simple way to provide corporate managers with development planning [31]. Intelligent financial data classification will not only improve the efficiency of financial personnel, but also formulate a long-term development plan for enterprises. This is a necessary step for enterprises to maintain long-term development in today economic globalization [32]. The SAS model library is a modeling application library proposed for intelligent finance. It contains functions such as data classification and data prediction. The algorithms basically included the current popular classification and forecasting algorithms, and there are a series of postprocessing function algorithms for data statistics, which is relatively easy for financial personnel to apply and build. The combination of the advantages of the SAS model and multisource data is a meaningful and necessary thing for the intelligent development of corporate finance.

### 2.2. Method and Source of Data Acquisition

**Figure 1:**
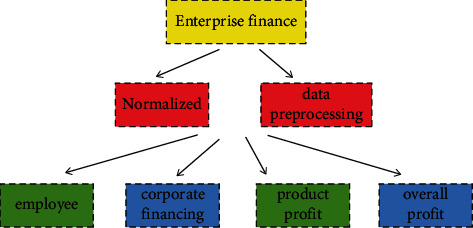
The processing flow of enterprise financial multisource data by SAS model.

For an enterprise, other financial information such as product information, employee information, and revenue performance generated every day is complex and multisource. In the today era, company financial information comes from multiple sources, such as the company employee performance information, employee bonus information, product marketing information, and corporate financing information. Not only are these sources of information different, but the proportions occupied and the contribution to the long-term development of an enterprise are also different. How to better fully dig out the relevance of these financial information, make better classification, and make predictions based on the classification information is meaningful for the development of an enterprise. If such a complicated corporate financial data only relies on financial personnel to classify and predict the development trend of the corporate economy, it will be a laborious task, and the accuracy rate will be difficult to guarantee. At the same time, the invisible correlation between these financial data is difficult to discover only by relying on the experience and knowledge of financial personnel. Artificial intelligence methods have been proven in many fields to discover the mapping relationships and correlations between high-dimensional and nonlinear data. For the application of the SAS model, this paper selects the employee performance, the corporate financing, the product sales, and the overall profit and loss of the company as multiple data sources for intelligent classification. Because this type of corporate financial data sources is complex, if the SAS model can better intelligently perform this type of financial data classification, this is easily extended to other areas of corporate finance.  Figure 1 shows the process of intelligent classification of enterprise financial multisource data through SAS model. Multisource data mainly includes employee performance, product marketing, corporate financing, and e-commerce marketing performance.

## 3. Intelligent Classification Method of Enterprise Financial Multisource Data

### 3.1. The Introduction of SAS Model

The SAS model is a commonly used data analysis model in financial management. It mainly refers to a comprehensive discipline model collectively referred to as financial analysis, data management, corporate economic analysis, and accounting analysis. It can give full play to its own advantages to sort out and summarize the past economic conditions of the enterprise, analyze its status quo, and predict its future development trend. It is also an important analysis model for the evaluation of enterprise profit and loss, investment recovery status, and profit and loss of marketing activities. It can be seen that its application range is relatively wide and it has also gained the trust of enterprises and financial personnel. The financial analysis of a company mainly includes corporate strategy analysis, accounting analysis, and financial statement analysis. The above three types of analysis are called SAS financial analysis. This article aims mainly to intelligently classify multisource financial data of enterprises and make effective predictions. Since the sources of corporate financial data are complex and diverse, these redundant data require intelligent algorithms to perform intelligent classification based on the internal connections of the data themselves. These classification algorithms are relatively easy to implement for the SAS model. At the same time, the SAS model can be used to classify the financial data of the enterprise, and then an effective evaluation and prediction can be made. The decision tree and clustering algorithm in the SAS model library have been well proven in the field of corporate finance. They have a good classification effect. The data in this paper is multisource data of corporate finance, which requires the SAS classification model.

### 3.2. The Introduction of Intelligent Classification Algorithm

**Figure 2:**
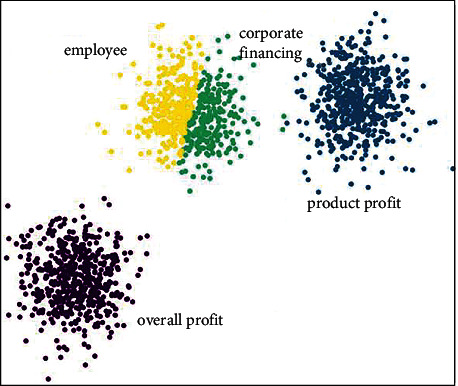
Classification of enterprise financial data using clustering methods.

The sources of enterprise financial data are diverse and complex. When only relying on the processing of financial personnel, the work is extremely complicated and prone to errors. At the same time, there are nonlinear high-dimensional correlations between multisource financial data, which makes it difficult for financial personnel to rely solely on professional knowledge. The recent rapid development of intelligent financial technology is a good thing for the development of financial personnel and enterprises. The SAS model is a commonly used model in the financial field. It can not only effectively classify financial multisource data, but also effectively predict the future trend of the enterprise economy, which is meaningful task to enterprise managers. There are many classification methods in the SAS system model, such as decision trees, support vector machines, logistic regression, and clustering. The schematic in Figure 2 shows the application of the clustering method from the SAS model to the intelligent classification of enterprise financial multisource data. It can be seen that clustering can classify four enterprise data sources according to different distances. The sample images were obtained from the website https://image.so.com.

Clustering is a type of machine learning method that can classify data based on the distance between data or the density of data. The standard for evaluating the quality of the classification results is that the distances between different types of data are as large as possible, and the distances or the differences between the densities of the same type of data are as small as possible. In this paper, the multisource financial data of enterprises needs to be divided into four categories: employee performance, product quality, corporate financing performance, and enterprise profit and loss. Therefore, the clustering method based on distance is adopted.

Equation (1) shows the expression of the external evaluation index, where *a*, *b*, *c*, and *d* represents employee performance, enterprise product profit and loss, enterprise financing, and overall enterprise profit and loss in enterprise financial multisource data.

(1) $$\begin{array}{c} R=\frac{a+d}{a+b+c+d}. \end{array}$$

Equation (2) shows the index of the Rand statistic, where *P* represents the precision rate and *R* represents the recall rate. *β* is a constant parameter; when *β*  = 1, this is the most common F1-measure.

(2) $$\begin{array}{c} F=\frac{\left(\beta^{2}+1\right)PR}{\beta^{2}P+R}. \end{array}$$

Equation (3) shows the FM parameters of external indicators, where *P* is the accuracy rate and *R* is the recall rate. The larger the FM value, the better the clustering effect of corporate finance.

(3) $$\begin{array}{c} \text{FM}=\sqrt{\frac{a}{a+b}}*\sqrt{\frac{a}{a+c}}=\sqrt{P*R}. \end{array}$$

Common measurement methods include Euclidean distance, Manhattan distance, and Chebyshev distance. These indicators evaluate the clustering effect from different angles. It is mainly aimed at the evaluation of samples, which is a more refined method of evaluation. Equation (4) shows the expression for the Euclidean distance, a measure of the distance between two points in space.

(4) $$\begin{array}{c} di\;ct_{e\;d}=\sqrt{\sum_{k=1}^{m}{\left(x_{ik}-x_{jk}\right)}^{2}}. \end{array}$$

Equation (5) shows the Chebyshev distance, an evaluation index of clustering effect, which defines a point in space as the difference between the absolute values of the coordinate values.

(5) $$\begin{array}{c} dict_{cd}=\underset{t⟶\infty }{\lim}{\left({\sum_{k=1}^{m}\left|x_{jk}-y_{ik}\right|}^{l}\right)}^{1/l}. \end{array}$$

Equation (6) shows the Minkowski distance evaluation index, which is a measure in Euclidean space, and also a variant extension of Euclidean distance and Manhattan distance. When *P* takes different values, it represents different distance evaluation criteria.

(6) $$\begin{array}{c} dict_{\min d}={\sqrt[{p}]{\sum_{k=1}^{m}\left|x_{ik}-y_{jk}\right|}}^{p}. \end{array}$$

### 3.3. Time Correlation Algorithm of Enterprise Financial Multisource Data

**Figure 3:**
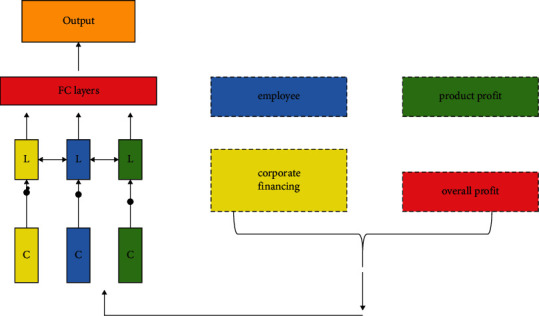
Prediction process of multisource financial data of enterprises.

After clustering, the multisource financial data of enterprises can be divided into four categories: employee performance, product quality, corporate financing performance, and overall corporate profit and loss. For an enterprise manager, this is not only concerned with the classification of enterprise multisource data. The future of economic development trend of enterprises is also the focus of attention for the business manager. Combining the characteristics of enterprise data itself, this paper adopts long short-term memory recurrent neural network to predict the future development trend of enterprise financial data. The advantage of long short-term memory neural network from convolutional neural network is that it can effectively map the temporal correlation between data. However, there is not only the correlation between categories, but also a certain time correlation between the data itself. The development of an enterprise is often related to policies in different time periods and the development trend of the global economy. Therefore, the long short-term memory recurrent neural network is used to predict the temporal correlation between the multisource financial data of the enterprise and intuitively show the prediction results to the enterprise decision makers, which will assist the enterprise to make decisions.  Figure 3 shows the prediction process of multisource financial data of enterprises.

Equation (7) shows the expression of the “forget” gate of the long short-term memory neural network, which selectively accepts part of the input at the previous moment and the input at this moment and combines them as the input value of the next gate structure. *ω* is the weights, *s*_*b*_^*t*−1^ is the state value of the last moment *t* − 1, and *x*_*i*_^*t*^ is the input parameter.

(7) $$\begin{array}{c} \alpha_{l}^{t}=\sum_{t=1}^{k}\omega_{il}x_{i}^{t}+\sum_{b=1}^{v}\omega_{ct}s_{b}^{t-1}. \end{array}$$

Equations (8) and (9) show the structure of the input gate. This layer mainly performs feature extraction for the input of the “forget” gate, and it undergoes nonlinear transformation through the activation function.

(8) $$\begin{array}{c} b_{\vartheta }^{t}=f\left(\alpha_{\vartheta }^{t}\right),f=\tanh, \end{array}$$

(9) $$\begin{array}{c} {\tilde{C}}_{t}=\tanh\left(w_{c}•\left[h_{t-1},P_{t}\right]+b_{c}\right). \end{array}$$

It can refresh the variable by the following formula:

(10) $$\begin{array}{c} {\overset{⟶}{b}}_{t}=f_{t}\times \overset{⟶}{b_{t-1}}+i_{t}\times {\overset{⟶}{b}}_{t}. \end{array}$$

Equations (11) and (12) show the output gate structure of the neural network structure, which is a layer for final feature input that selectively inputs temporal features and combines the state information of different historical moments and the feature information of the input gate.

(11) $$\begin{array}{c} s_{c}^{t}=b_{\vartheta }^{t}s_{c}^{t-1}+b_{i}^{t}g\left(a_{c}^{t}\right), \end{array}$$

(12) $$\begin{array}{c} {\overset{⟶}{h}}_{t}=O_{t}\times \;\tanh\left({\overset{⟶}{C}}_{t}\right). \end{array}$$

### 3.4. The Preprocessing of Multisource Financial Data of Enterprise

The data preprocessing stage is an important stage for using the SAS model. This is because the sources of corporate financial data are different and there are certain gaps in characteristics. The purpose of data preprocessing is to normalize different financial data sources so that these data conform to the same distribution and are in the same order of magnitude, which is beneficial for both classification algorithms and enterprise time series predictions. If these multisource financial data are not preprocessed, this will cause uneven weight distribution in the classification and prediction process, which will not only reduce the learning ability of the model but also reduce its generalization ability. In other words, this model may show better accuracy only for specific corporate finances and will produce poor results when forecasting and categorizing the financial multisource data of other companies. From the above description, it can be learned that the preprocessing of multisource financial data is not only an important step of the SAS model, but also an important step to improve the accuracy and generalization ability of the model. In this study, multisource financial data will be processed into distribution characteristics that conform to the normal distribution, and the data will be processed into an order of magnitude between 0 and 1. The standard processing methods are adopted in this paper to preprocess multisource enterprise data.

## 4. Classification and Predictive Analysis of Enterprise Financial Multisource Data

**Figure 4:**
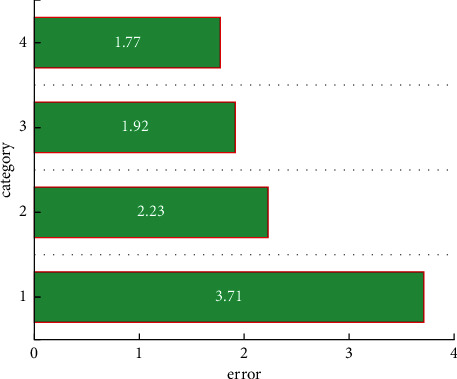
The classification error of multisource financial data.

**Figure 5:**
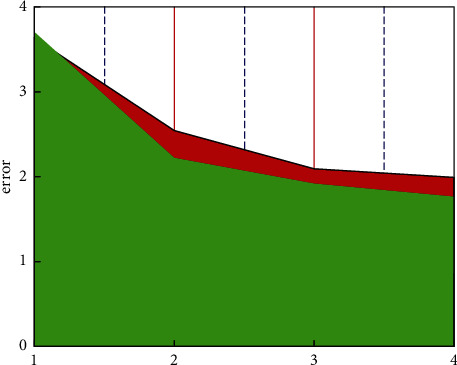
Forecast and actual development trend of corporate financial data.

This research first analyzes the results of intelligent classification of the enterprise's multisource financial data. It can be clearly seen from Figure 4 that the errors of the classification results are all within 4%, which is a trustworthy result for enterprise managers to make decisions. This article mainly classifies employee performance, product marketing, corporate financing rate, and overall profit and loss. It can be seen that the smallest classification error is only 1.77%, which is an acceptable error for a large enterprise. This part of the error is mainly from employee performance. The reason why this part is the smallest is mainly that the performance of employees is within a controllable range. The largest error is 3.77%. This part of the error comes from the overall profit and loss of the enterprise. The reason for this part of the error is that the overall profit and loss of the enterprise is a complex and changeable variable, and there are certain differences between it and these multisource data. Part of the correlation also comes from accumulated errors. Generally speaking, this model is credible in the intelligent classification of enterprise financial multisource data.  Figure 5 reflects the forecast and actual development trend of multisource financial data of enterprises. For these four types of classified multisource data, the overall forecast trend is consistent with the actual development trend of the company, but there is a small error. From a macro perspective, the difference between the development trend of corporate finance and the multisource data is better mapped, which shows that this model has certain accuracy in the prediction of corporate multisource finance. For the prediction of employee performance, there is basically no error, but for the other three predictions, there are certain errors. This is mainly due to the sudden change of corporate finance over time.

**Figure 6:**
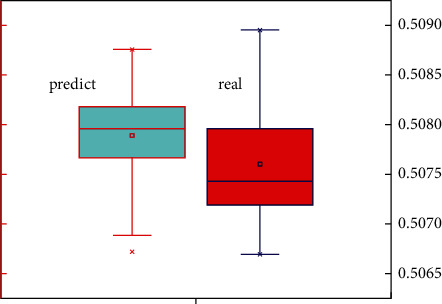
Box diagram of forecast and real value of enterprise financial multisource data.

**Figure 7:**
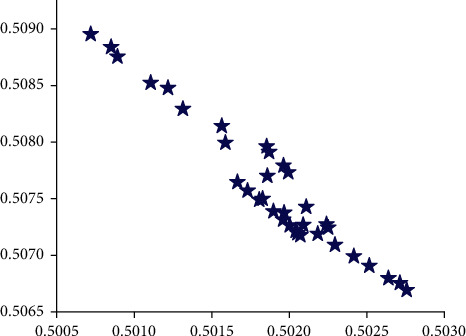
Linear correlation of multisource data of enterprise finance.

In order to further analyze the accuracy and feasibility of the SAS model in the classification and forecasting of corporate financial data, Figure 6 shows a box diagram of the forecasted value of financial multisource data and the development trend of actual data. It can clearly indicate not only the trend and accuracy of the predicted value, but also the overall distribution trend of the predicted value and the actual value. From the overall point of view in Figure 6, the predicted value of corporate financial data is generally higher than the actual data, but the error between them is relatively small. At the same time, the predicted value of financial data is relatively more concentrated than the actual financial data, which is mainly caused by the concentrated distribution of weights. It can be seen from Figure 6 that the predicted value of the multisource data of corporate finance and the actual value are basically between 0.506 and 0.509, and the error is relatively small.  Figure 7 shows the linear correlation between the predicted value of corporate financial multisource data and the actual data, which can show the stability of the predicted value. It can be seen from Figure 7 that the data points are distributed on both sides of the linear function and that the distance from the straight line of the linear function is relatively small, which shows that the forecast of multisource financial data maps the actual data value well. It can also be seen from the figure that only a small part of the data deviates from the linear function and the distance is relatively large. This may be due to the large randomness of this type of data source and the obvious relationship with time, which requires the increase of this part of the data to improve the accuracy of this part of the forecast and classification.

**Figure 8:**
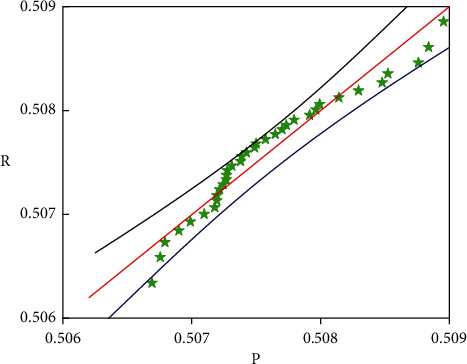
Predictive distribution of corporate financial data.

**Figure 9:**
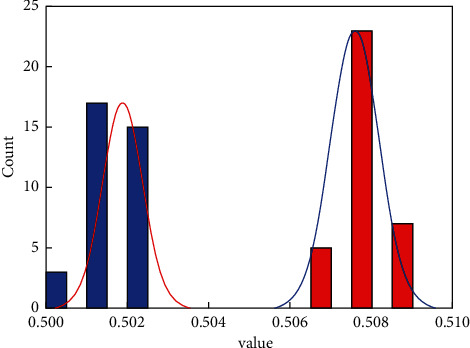
Cumulative error distributions for two types of financial data.

Figure 8 shows the normal distribution of errors for multisource corporate financial data. It can be seen that the distribution of multisource financial data errors is a relatively average one, and the data points are all within the upper and lower boundaries of the normal distribution. Only some of the financial data points are off the center line, but the error in this part of the data is also within an acceptable range. The correlation coefficients all exceed 0.96, which shows that the financial data of enterprises has a good prediction effect. In addition, the data points are basically distributed on both sides of the *y* = *x* function, which means that there is a good correlation.  Figure 9 shows the forecast classification error trends for the two types of financial data sources. It can be seen that the errors fit the normal distribution curve well, which is the same as the input financial data source in the preprocessing process. Moreover, the distribution of the above two types of financial data with large errors is relatively uniform, which means that the SAS model is suitable for the classification and prediction of multisource financial data of enterprises.

## 5. Summary of Research on Intelligent Classification of Enterprise Financial Data

The sources of corporate financial data are often diverse, complex, and changeable due to the development trend of economic globalization and the continuous stimulation of the e-commerce economy. It would be a laborious and error-prone task to rely solely on financial staff to organize and forecast a company's multisource financial data. SAS model is a model specially used for enterprise financial analysis and prediction. It can quickly and accurately realize intelligent classification of financial multisource data, and the efficiency is high.

This paper firstly uses clustering to effectively classify employee performance, product profit and loss, corporate financing, and overall profit and loss and uses the classification results to accurately predict the financial development trend of the company. In general, the classification accuracy is relatively high, the largest error is only 3.71%, and the smallest error is only 1.77%. The error is largest compared to other factors, which is due to the large randomness of the overall profit and enterprise loss, and it is also affected by the changes of socioeconomy. The other three types of financial data have cumulative errors. After a reasonable classification by the SAS model, these classified data are used to predict the future development trend of the enterprise finance. Generally speaking, the financial forecast of the enterprise is in good agreement with the development trend and change trend of the actual data value, which is a trustworthy model for the enterprise. The linear correlation between them is also relatively good. The data points are distributed on both sides of the linear function, and the distance from the linear function is relatively close, which shows that this model is feasible in the classification and prediction of enterprise financial intelligence.

## Data Availability

The dataset can be accessed upon request.
